# The potential role of GLP-1 receptor agonists in the management of psoriatic disease: a scoping review

**DOI:** 10.1007/s00011-025-02140-2

**Published:** 2025-11-21

**Authors:** Simona Buonanno, Carla Gaggiano, Riccardo Terribili, Luca Cantarini, Bruno Frediani, Stefano Gentileschi

**Affiliations:** Rheumatology Section, Department of Medical Sciences, Surgery and Neuroscience, Siena University Hospital, University of Siena, Policlinico “Le Scotte”, Viale Mario Bracci 16, 53100 Siena, Italy

**Keywords:** Psoriatic disease, Psoriasis, Glucagon-like peptide-1 receptor agonists, Obesity, Type 2 diabetes mellitus, Cardiovascular disease

## Abstract

**Background:**

Psoriatic disease (PsD) is a chronic systemic inflammatory condition associated with significant cardiometabolic comorbidities, including obesity, type 2 diabetes mellitus (T2DM), and cardiovascular (CV) disease. These comorbidities are interlinked via shared immunopathogenic mechanisms, notably chronic low-grade inflammation driven by Th1/Th17 cytokines such as TNF, IL-6, and IL-17. Obesity, in particular, exacerbates PsD severity and treatment resistance, underscoring the need for integrated therapeutic strategies. This scoping review investigates the biological rationale and evidence for the use of glucagon-like peptide-1 receptor agonists (GLP-1RAs) in PsD.

**Findings:**

Originally developed for T2DM, GLP-1RAs have demonstrated efficacy in reducing weight and improving glycemic control and CV outcomes. Evidence also suggests immunomodulatory properties through modulation of key inflammatory pathways and immune cell activity. We examined studies addressing: (1) the impact of obesity, T2DM, and CV disease on PsD; (2) outcomes of GLP-1RAs in these comorbidities; and (3) their potential in related rheumatologic and dermatologic diseases. GLP-1RAs show promise in reducing PsD burden by improving metabolic parameters and reducing systemic inflammation. Early clinical and preclinical data suggest benefits also in rheumatoid arthritis, osteoarthritis, osteoporosis, psoriasis, and hidradenitis suppurativa.

**Implications:**

GLP-1RAs represent a novel, multifaceted therapeutic option in PsD, targeting both metabolic and inflammatory components. Further clinical trials are warranted to define their role in comprehensive PsD management and validate their disease-modifying potential.

**Supplementary Information:**

The online version contains supplementary material available at 10.1007/s00011-025-02140-2.

## Introduction

Psoriatic arthritis (PsA) is a chronic inflammatory disease that affects skin, nails, peripheral and axial joints, as well as enthesis. Its prevalence is estimated to range from 0.1 to 1% in the general population and reach approximately 20% among individuals with psoriasis (PsO) [1].

The significant overlap between PsA and PsO, with PsA manifesting in up to 41% of PsO cases, has given rise to the concept of “Psoriatic Disease” (PsD). This term reflects the recognition of a systemic condition that extends beyond the skin and musculoskeletal system, encompassing shared pathogenetic mechanisms, diverse clinical manifestations, and associated comorbidities [2].

PsO and PsA are notably associated with increased concentrations of inflammatory cytokines, including tumor necrosis factor (TNF), interferon (IFN)-γ, interleukin (IL)-17, IL-22, and IL-23, in both skin and synovial tissues. This elevation results from the activation of inflammation driven primarily by Th1 and Th17 pathways [3].

PsD carries a significant burden of cardiometabolic comorbidities which are more prevalent compared to the general population [4, 5]. Among these, obesity (defined by the WHO as a body mass index (BMI) ≥ 30 kg/m^2^) [6], is characterized by low-grade inflammation driven by adipokine secretion from adipose tissue. This condition is more prevalent in PsA patients compared to the general population and significantly contributes to their cardiovascular (CV) burden [7, 8]. Hypertension, diabetes, dyslipidemia, obesity, metabolic syndrome, and CV events share common immunopathogenic mechanisms tied to systemic inflammation and are closely linked to PsA disease severity and extent. Additionally, they can impact the efficacy of treatments in achieving disease control [3, 9, 10].

Addressing low-grade inflammation associated with obesity, reducing cardiovascular burden, and achieving overall health improvement remain significant unmet needs in PsD. In this context, the emergence of new molecules capable of effectively treating obesity and associated metabolic comorbidities offers a valuable opportunity for broadening therapeutic strategies in PsD patients.

Glucagon-like peptide-1 receptor agonists (GLP-1RAs) are a class of drugs that targets incretin hormone pathways. GLP-1 receptor (GLP-1R) is broadly expressed in various tissues, including nerves, pancreatic islets, heart, lungs, skin, and other organs [11]. GLP-1RAs, originally developed for the treatment of type 2 diabetes mellitus (T2DM), demonstrated efficacy in promoting weight loss in both preclinical and clinical studies [12]. Recent research has identified GLP-1R expression on innate and innate-like immune cells, indicating a potential role for GLP-1RAs in regulating immune responses among various inflammatory diseases [13].

This scoping review aims to explore the biological rationale and the existing evidence supporting the application of GLP-1RAs in the management of PsD.

## Research methodology

Our methodological approach follows the guidelines outlined in the PRISMA 2020 Statement extensions for scoping reviews. A comprehensive database search was conducted in PubMed, MEDLINE, Scopus, Embase, and Google Scholar covering the period from inception (1996, 1966, 2004, 1947 and 2004, respectively) to September 2025. The inclusion criteria encompassed English-language publications or publications with an English translation available, including conference abstracts, observational studies, randomized controlled trials (RCTs) and meta-analyses.

We conducted our research focusing on three main topics:

1. The impact of obesity, T2DM, and CV events on PsD.
2. The outcomes of GLP1-RAs in PsD comorbidities such as obesity, T2DM, and CV events.
3. The potential role of GLP1-RAs in rheumatologic diseases (including arthritis, gout, osteoarthritis, and osteoporosis) as well as autoimmune skin diseases (such as psoriasis and hidradenitis suppurativa) sharing common pathophysiological pathways with PsD.

The search terms were constructed by combining keywords and MeSH related to psoriatic arthritis, psoriasis, rheumatoid arthritis, osteoarthritis, osteoporosis, hidradenitis suppurativa, GLP-1RAs, obesity, T2DM and CV risk factors.

The study selection consisted of two phases: evaluation of titles/abstracts and full-text review. Data was extracted using a standardized form that included the following information: author, year and country of study, study design and population, key outcomes and results, main conclusions. Studies were then selected based on the relevance of their subject matter to the three topics identified above. Data were synthesized in a narrative form and supplemented with tables and figures. Although the scoping review does not include a formal risk of bias assessment, a critical discussion of the methodological quality of the included studies was conducted.

## Results

### The interconnection between psoriatic disease and obesity, type 2 diabetes mellitus, and cardiovascular events

Inflammation in PsD plays a pivotal role in exacerbating atherosclerosis and cardiometabolic diseases, with proinflammatory cytokines such as TNF, IL-1, IL-6, and IL-17 contributing to endothelial dysfunction and vascular damage. It is known that chronic activation of Th1 and Th17 pathways leads to increased levels of these mediators in PsO and PsA, promoting a proatherogenic phenotype. Furthermore, metabolic syndrome (MetS) in PsD is linked to systemic inflammation, with obesity, insulin resistance, and adipokines further aggravating metabolic imbalances [14]. The process by which systemic inflammation, obesity, and metabolic abnormalities collectively promote insulin resistance and endothelial dysfunction driving to atherosclerosis and ultimately in CV disease development, is described by the “psoriatic march” hypothesis [15].

### Obesity and PsD

**Fig. 1:**
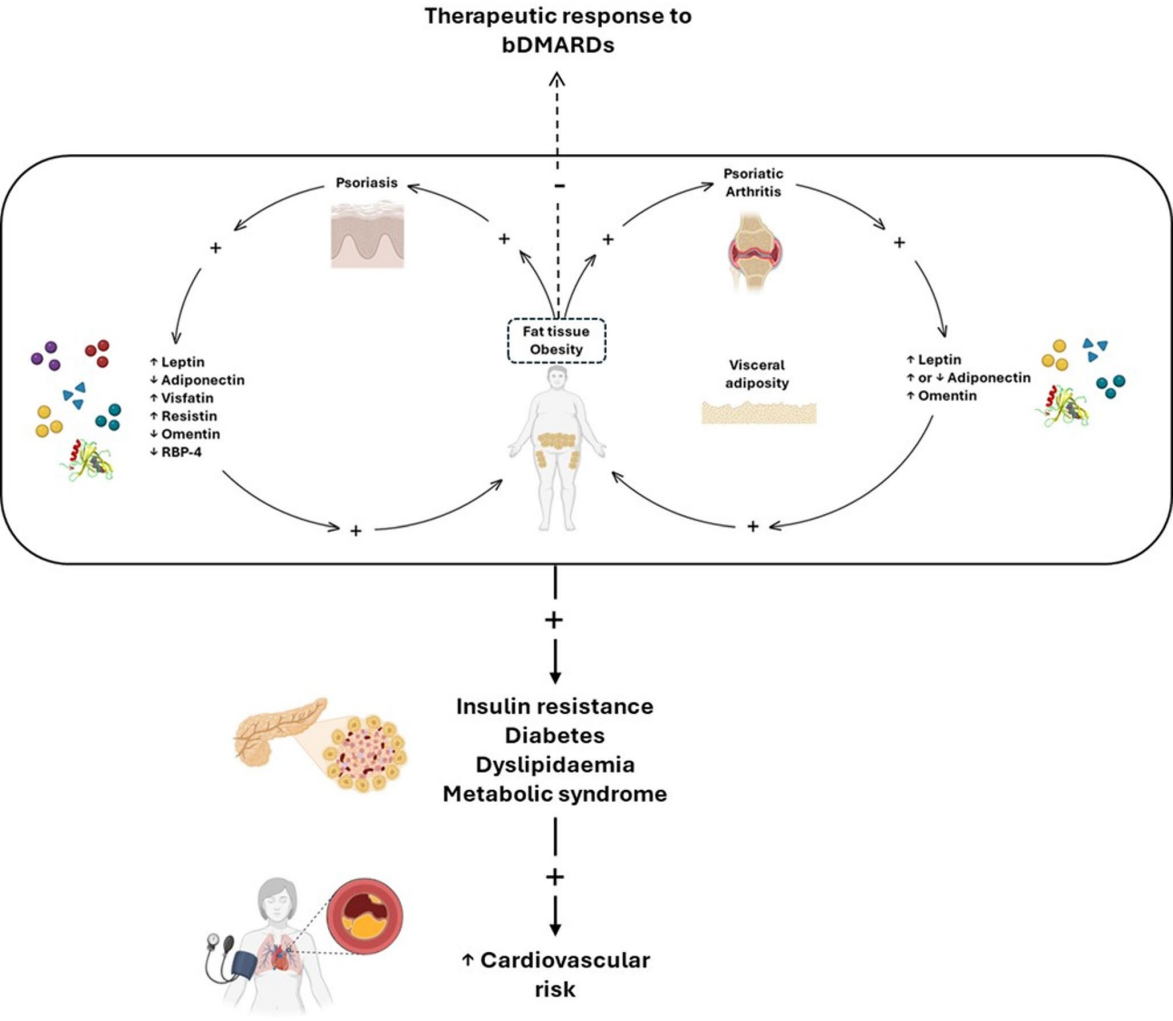
The interconnection between psoriasis (PsO), psoriatic arthritis (PsA) and obesity and its impact on cardiovascular health. Obesity is a risk factor PsO and PsA, both linked to complications like metabolic syndrome, dyslipidemia, diabetes, and insulin resistance, which increase cardiovascular risk. In PsA patients, excessive abdominal fat worsens body composition, contributing to metabolic syndrome and cardiovascular diseases. Adipokines, such as leptin and adiponectin, are involved in these complications, with altered levels potentially worsening inflammation, insulin resistance, and reducing cardiovascular protection. Additionally, excess fat may reduce the effectiveness of biological treatments, and anti-tumor necrosis factor (TNF) agents are associated with weight gain. *bDMARDs* Biologic disease-modifying anti-rheumatic drugs; *RBP-4* Retinol binding protein 4

**Fig. 2:**
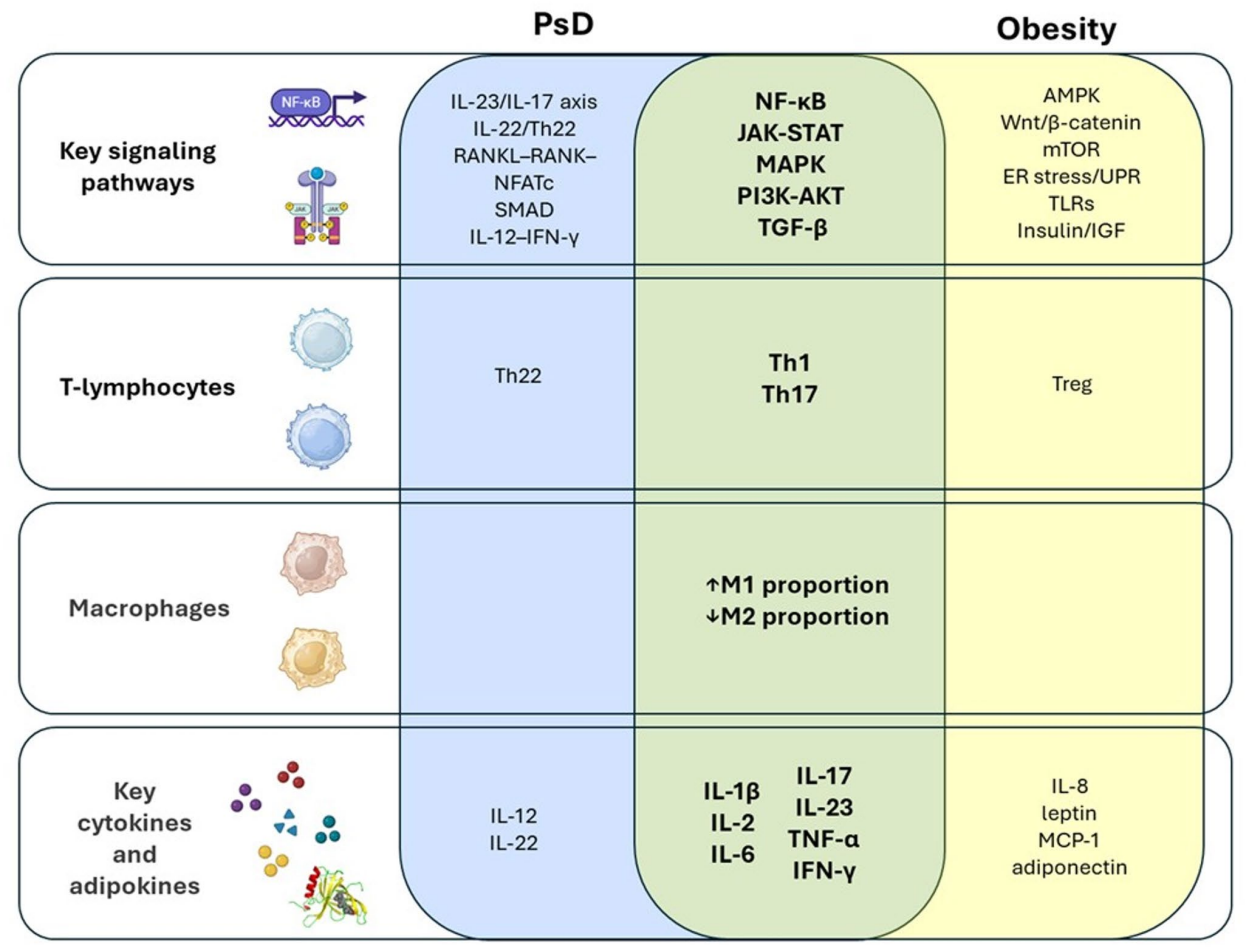
Shared molecular and immunological pathways in obesity and psoriatic disease. Shared pathways between PsD and obesity highlighted in bold. *AKT* known as the *PKB* Protein Kinase B; *AMPK* AMP-activated protein kinase; *ER* Endoplasmic Reticulum; *IGF* Insulin-like Growth Factor; *JAK-STAT* Janus kinase-signal transducer and activator; *MAPK* Mitogen-activated protein kinase; *MCP-1* Monocyte chemoattractant protein-1; *mTOR* Mechanistic target of rapamycin; *NFATc* Nuclear Factor of Activated T-cell cytoplasmic component; *NF-κB* Nuclear factor kappa B; *PI3K* Phosphatidylinositol 3-kinase; *PsD* Psoriatic disease; *RANK* Receptor Activator of Nuclear Factor Κb; *RANKL* Receptor Activator of Nuclear Factor κB Ligand; *SMAD* SMA small worm mutant and MAD Mothers against decapentaplegic; *TGF-β* transforming growth factor-β; *TLRs* Toll-like receptors; *UPR* Unfolded Protein Response; *Wnt* Wingless/Int-1

Studies show a strong association between increased adiposity and the risk of PsO and PsA. Higher BMI and central obesity significantly increase the likelihood of developing PsO and PsA, with severe obesity amplifying this risk [16]. Also, the association between obesity and PsA is potentially bidirectional, as patients with joint dysfunction are often less physically active [17, 18]. Obesity increases joint load, alter their mechanics, and lead to repetitive micro-trauma, which, besides causing OA, may also trigger inflammation in joints and enthesis [19–22]. In addition, adipose tissue, through the secretion of adipokines, plays a central role in immune and metabolic processes. Leptin, visfatin, and resistin contribute to inflammation, while adiponectin (especially its low molecular weight isoform), has anti-inflammatory effects. Adipose tissue also directly produces pro-inflammatory cytokines such as IL-6, TNFα, and IL-8. Additionally, adipokines influence metabolic functions, including insulin sensitivity, linking adipose tissue to both immune regulation and metabolic health (Fig. 1). Figure 2 provides a synthesis of the molecular and immunological pathways shared by obesity and PsD, further pointing out the central role of adipokines in these conditions [23–31].

In a 2017 review, Kruglikov et al. emphasize the role of white adipose tissue (WAT) in PsO and PsA [32]. They identify an inflammatory phenotype in WAT nearby PsA affected joints, such as Hoffa's fat pad and areas around the heel, elbow, tendons, and bursae [33–35]. This metabolically active WAT produces interleukins, adipokines, antimicrobial peptide (AMP) cathelicidin [36, 37] and possibly collagen type VI (COL6) contributing to disease mechanisms [38–47]. In PsA patients, WAT localized in entheses-associated regions like the retrocalcaneal fat pad, shows impaired lipolysis, reduced adiponectin secretion, and increased fatty acid β-oxidation, possibly emphasizing its pathological significance [48–50].

In this context, weight loss represents a valuable complementary strategy for modifying PsA, encompassing dietary changes, exercise, as well as emerging weight-loss treatments, such as GLP1-RAs and sodium-glucose co-transporter 2 inhibitors (SGLT2i) in patients with concomitant MetS [51]. Gratacós et al. link obesity to an increased risk of treatment failure in PsA [52], particularly with anti-TNF drugs, with obese patients showing a lower probability of achieving and maintaining minimal disease activity (MDA). Obesity-related low-grade inflammatory state can alter the pharmacokinetics of anti-TNF agents, leading to reduced drug concentrations and shorter half-life. Therefore, obese patients may require dose adjustments, though supporting evidence for this approach is limited [53–55]. Recently, IL-12/IL-23 inhibitors have been introduced as a new class of drugs for PsA management, with evidence suggesting that their efficacy is less influenced by body weight. Ustekinumab, an IL-12/IL-23 inhibitor, has shown promising results, providing effective and sustained responses in PsA patients [2, 19]. Weight loss significantly improves response rates: a 5–10% weight loss increases the odds of achieving MDA (OR 3.75), with over 10% weight loss raising it further (OR 6.67) [56].

### Type 2 diabetes mellitus and PsD

Patients with PsA exhibit an increased prevalence of T2DM compared to the general population with a particularly enhanced risk in women [57] and those with active disease [58]. Late-onset PsO (after 40 years) further elevates DM risk [59]. Both PsA and PsO are linked to insulin resistance, but PsA appears more strongly associated with DM [60]. The pathogenic link between PsA and T2DM is not yet fully understood, but inflammatory cytokines, particularly TNF and IL-17, are known to impair insulin signaling and promote insulin resistance [61]. In particular, TNF exacerbates insulin resistance by inhibiting insulin receptor phosphorylation and GLUT4 translocation [62]. Adipokines, such as adiponectin and omentin, are also involved, with decreased levels contributing to metabolic dysfunction. Genetic studies have not identified a direct PsA-DM association, thus inflammation-driven metabolic changes remain key contributors. Targeted therapies, including TNF inhibitors, may improve both PsA and metabolic parameters [63].

### Cardiovascular events and PsD

A meta-analysis of 11 studies reported a 43% increased risk of CV diseases in PsA patients compared to the general population, with elevated risks of myocardial infarction (68%), cerebrovascular disease (22%), and heart failure (31%) [64]. Disease activity markers, including polyarthritis, dactylitis, and systemic inflammation, were linked to higher CV event risk, and atherosclerotic plaque burden correlated with PsA severity. PsO, particularly in severe cases, was identified as an independent risk factor for ischemic heart disease, stroke, and CV mortality [65–67], but the incidence of cerebrovascular disease and metabolic disorders appears higher in those with PsA [68, 69]. The risk of major adverse CV events (MACE) in PsA patients not using disease-modifying antirheumatic drugs (DMARDs) was comparable to that in rheumatoid arthritis (RA) after adjusting for traditional CV risk factors [70].

The American College of Cardiology (ACC) / American Heart Association (AHA) guidelines for primary CV disease prevention recognize psoriasis and other chronic inflammatory conditions as risk-enhancing factors, recommending more aggressive management. Consequently, a comprehensive CV risk assessment and a multidisciplinary management are essential for patients with PsA [19, 71].

## Evidence of the role of GLP-1RAs in managing psoriatic disease comorbidities

GLP1-RAs play a pivotal role in managing comorbidities associated with PsD including T2DM, obesity and CV events. Several molecules are currently available on the market, with partially different mechanisms of action, indications, dosages, and routes of administration (Table 1).

**Table 1:** Overview of GLP-1RAs licensed for the treatment of type 2 diabetes mellitus

Molecule	Brand name	Mechanism of action	License date	Indications	Device/Route	Posology
Exenatide	Byetta ©, Bydureon ©	GLP-1 receptor agonist, stimulates glucose-dependent insulin release and reduces appetite	2005: Byetta © 2012: Bydureon©	T2DM	*Pen*; Subcutaneous injection	Byetta ©: 5–10 mcg twice daily Bydureon ©: 2 mg once weekly
Liraglutide	Victoza ©, Saxenda ©	GLP-1 receptor agonist, slows gastric emptying and reduces appetite	2010: Victoza © 2014: Saxenda©	T2DM: Victoza© Obesity: Saxenda©	*Pen*; Subcutaneous injection	Victoza ©: up to 1.8 mg/day Saxenda ©: up to 3 mg/day
Dulaglutide	Trulicity ©	Long-acting GLP-1 receptor agonist	2014	T2DM	*Pen*; Subcutaneous injection	0.75–1.5 mg once weekly
Lixisenatide	Adlyxin © (US) Lyxumia © (EU)	GLP-1 receptor agonist, short-acting, enhances glucose-dependent insulin secretion	2016	T2DM	*Pen;* Subcutaneous injection	10 mcg once daily for 14 days, then 20 mcg once daily
Semaglutide	Ozempic ©, Rybelsus ©, Wegovy ©	GLP-1 receptor agonist, reduces appetite and body weight	2017: Ozempic© 2019: Rybelsus© 2021: Wegovy ©	T2DM: Ozempic ©, Rybelsus© Obesity: Wegovy ©	*Pen*; Subcutaneous injection: Ozempic© Wegovy © *Tablets*; Oral Rybelsus©	Ozempic ©: up to 2 mg/week Rybelsus © (oral): up to 14 mg/day Wegovy ©: up to 2.4 mg/week
Tirzepatide	Mounjaro ©	Dual GLP-1/GIP agonist, enhances insulin secretion and appetite reduction	2022	T2DM Obesity	*Pen*; Subcutaneous injection	Initial dose 2.5 mg/week, increasing up to 15 mg/week

*EMA* European Medicines Agency; T2DM: type 2 diabetes mellitus

### Outcomes in diabetes mellitus

GLP-1, a member of the incretin hormone family secreted in response to nutrient intake, enhances insulin secretion in a glucose-dependent way [72]. Acting as analogs of endogenous GLP-1, GLP-1RAs have emerged as one of the most effective therapeutic options for managing T2DM due to their multifaced effects on glucose metabolism (Table 2) [73].

**Table 2:** The multifaceted role of GLP-1 in glucose regulation and its therapeutic benefits in type 2 diabetes mellitus management [73]

Antidiabetic effect	Mechanism of action	Clinical Benefit
Glucose-dependent insulin secretion	Stimulates pancreatic β-cells to release insulin only in the presence of elevated glucose levels	Reduces hyperglycemia while minimizing the risk of hypoglycemia
Inhibition of glucagon secretion	Suppresses glucagon release from pancreatic α-cells during hyperglycemia	Decreases hepatic glucose production, improving fasting and postprandial glucose levels
Delayed gastric emptying	Slows gastric motility and nutrient absorption	Reduces postprandial glucose excursions
Increased β-cell survival	Promotes β-cell proliferation and reduces apoptosis	Helps preserve pancreatic function over time
Appetite suppression	Acts on the central nervous system (hypothalamus) to promote satiety	Aids in weight management, beneficial for insulin sensitivity
Reduction in hepatic glucose production	Indirect effect through suppression of glucagon and improvement in insulin sensitivity	Contributes to better glycemic control

GLP-1RAs, including exenatide, albiglutide, dulaglutide, and lixisenatide, demonstrated remarkable efficacy in improving glycemic control by lowering postprandial glucose levels and HbA1c. Exenatide lowers HbA1c and postprandial glucose, with the once-weekly regimen demonstrating greater efficacy compared to the twice-daily option [74]. Dulaglutide demonstrated superior glycemic control compared to exenatide and insulin glargine in the AWARD studies, with greater HbA1c reductions and improved patient satisfaction while it was noninferior to liraglutide in HbA1c reduction [75]. Lixisenatide, compared to liraglutide, significantly reduced postprandial glucose and lowered HbA1c by − 0.32% with a more favorable tolerability profile, particularly regarding gastrointestinal side effects. In contrast, liraglutide was more effective in reducing fasting glucose (− 0.51) [76]. In the SUSTAIN 1–5 and 7 trials, once-weekly subcutaneous semaglutide consistently reduced HbA1c, improved Fasting Plasma Glucose and self-monitoring blood glucose profiles with a low risk of hypoglycemia in patients with T2DM. The safety profile of semaglutide was comparable to other GLP-1RA [77]. In the SURPASS trials, once weekly tirzepatide (5–15 mg) significantly reduced HbA1c (1.87–3.02%) and improved several cardiometabolic risk factors. Tirzepatide demonstrated superior efficacy compared to placebo and other common glucose-lowering therapies, including semaglutide 1 mg, dulaglutide, insulin degludec, and glargine. All doses of tirzepatide were well tolerated, with a side-effect profile similar to that of GLP-1RAs [78].

### Outcomes in obesity

Among GLP-1RAs, liraglutide, semaglutide, and tirzepatide are licensed for the treatment of obesity. However, other agents in this class, including exenatide and dulaglutide, have shown effectiveness in inducing weight loss. Body weight reduction associated with this class of drugs is primarily due to a decrease in caloric intake rather than an increase in energy expenditure. The suppression of food intake can be attributed to three key factors: increased satiation, reduced appetitive drive, and visceral malaise after large meals in a minority of subjects [79]. A meta-analysis of 21 trials showed weight loss of 0.2 to 7.2 kg with GLP-1RAs, with higher doses leading to greater weight loss. Compared to pioglitazone and insulin glargine, which caused weight gain, patients on GLP-1RAs lost 4–5 kg [80]. Weight loss benefits are thought to be due to suppressed appetite, reduced body fat, and improved endothelial function [81].

Several clinical trials evaluated the effectiveness of GLP-1RAs in inducing weight loss in individuals with obesity, with or without concomitant T2DM. Evidence comes from large-scale studies such as the SCALE and LEAD trials for liraglutide, the STEP 1–8 program for semaglutide, the SURMOUNT 1–4 trials for tirzepatide, AWARD-11 for dulaglutide, and DURATION-1 for exenatide (Table 3).

**Table 3:** Summary of weight loss outcomes with GLP-1RAs in obesity management

Drug	Dose/Trial	Population	Duration	Average WL	≥ 5% WL (%)	≥ 10% WL (%)	Key comparisons
Liraglutide	3.0 mg/day (SCALE)[83]	Non-diabetics with BMI ≥ 30 or ≥ 27 + comorbidities	56 weeks	− 8.4 kg *vs.* − 2.8 kg (placebo)	63.2% vs 27.1% (placebo)	33.1% vs. 10.6% (placebo)	Effect confirmed in LEAD trials also at 1.8 mg. [84] STEP 8: inferior to semaglutide 2.4 mg
Semaglutide	2.4 mg/week (STEP 1–5, 8) [84–87]	Overweight/ obese ± T2DM	Up to 104 weeks	Up to − 17.4% (STEP 4)	> 85% in STEP trials	Up to 33–75%	Effect sustained with continued use (STEP 4) Superior to liraglutide 3.0 mg (STEP 8)
Tirzepatide	5, 10, 15 mg/week (SURMOUNT 1–4) [88–91]	Obese ± T2DM	72 weeks	− 15% (5 mg), − 19.5% (10 mg), − 20.9% (15 mg)	79–83% (T2DM: SURMOUNT-2)	51–57% (SURMOUNT-1)	Greater effect than all other GLP-1RAs. Effect maintained in SURMOUNT-4
Dulaglutide	1.5–4.5 mg/week (AWARD-11) [75]	T2DM, ± obesity	36 weeks	− 4.6 kg (4.5 mg) *vs* − 3.0 kg (1.5 mg)	Not reported	Not reported	Dose-dependent effect. Modest weight loss compared to semaglutide/ tirzepatide
Exenatide	2 mg/week (DURATION-1) [92]	T2DM	52 weeks	> 4 kg	Not reported	Not reported	HbA1c improvement. Less effective than semaglutide (56-week head-to-head trial)

*HbA1c* Glycosylated hemoglobin; *T2DM* Type 2 Diabetes Mellitus; *WL* Weight loss

A recent network meta-analysis including 27 RCTs and 15,584 participants evaluated efficacy and safety of seven GLP-1 RAs and polyagonists (GLP-1/GIP or GLP-1/GIP/Glucagon) for weight management in individuals with overweight or obesity. Greater weight reduction was observed in individuals with higher baseline BMI and in those with longer treatment cycles. Safety analyses indicated a higher incidence of adverse events in patients without T2DM, but no increased risk of serious or hypoglycemic events was identified across interventions. Overall, dual and triple receptor agonists (like tirzepatide and retatrutide) appear to achieve superior weight loss outcomes compared to other GLP-1 RAs [82].

### Outcomes in cardiometabolic risk

GLP1R expression has been detected at low levels in human atria and ventricles, both in cardiomyocytes and endothelial cells, though its precise localization remains unclear due to limitations in antibody specificity and differences at species level [93]. Cardiovascular outcome trials (CVOTs) with GLP-1RAs evaluated seven agents, with liraglutide, semaglutide, and dulaglutide showing significant CV and renal benefits. Liraglutide reduced CV mortality and myocardial infarction risk, while semaglutide and dulaglutide lowered non-fatal stroke incidence [94–96]. The REWIND study highlighted dulaglutide’s role in both primary and secondary CV prevention, making it the first Food And Drug Admininstration (FDA)-approved GLP-1RA for reducing MACE in T2DM patients [97]. In addition, clinical trials of GLP-1RAs assessed CV safety as secondary endpoints, including, blood pressure (BP), heart rate (HR), and CV events (arrhythmia, heart failure, myocardial infarction) [98].

#### Blood pressure

GLP1R agonists lower BP, particularly in hypertensive individuals, with reductions of 2–6 mmHg observed in clinical trials. These effects, independent of glucose lowering and weight loss, may contribute to CV benefits but are unlikely to fully explain MACE reductions. Preclinical studies confirm BP-lowering effects in hypertensive mice, potentially mediated by increased atrial natriuretic peptide secretion and improved endothelial function [81]. However, inconsistencies in human studies highlight the need for further research on GLP1R expression and vascular function [93].

#### Lipid profile

GLP-1RAs exert anti-inflammatory effects in the CV system, reduce hepatic steatosis, and lower circulating triacylglycerol and low-density lipoprotein (LDL) cholesterol levels. These effects, partially mediated by GLP1R + endothelial and intrahepatic γδ T cells, contribute to cardio-protection in T2DM patients [93].

#### Atherosclerosis

GLP-1RAs may exert anti-atherogenic effects, as CVOTs show reduced MACE after 12–18 months, particularly in patients with established CV disease. Preclinical studies in mice demonstrate reduced atherosclerotic plaque progression and increased plaque stability. These effects are partly mediated by reduced systemic inflammation, with decreased expression of TNF, Monocyte Chemoattractant Protein (MCP)-1, and IL-6 in macrophages. The precise mechanism remains unclear, but inter-organ communication, possibly via neural pathways, may contribute to GLP-1RA-mediated CV protection [93].

#### Myocardial infarction

In patients undergoing percutaneous interventions, GLP-1RAs may reduce myocardial infarction incidence and infarct size while improving left ventricle ejection fraction. Preclinical studies show GLP-1RAs mitigate ventricular dilation, fibrosis, and hypertrophy via insulin-like growth factor (IGF)-1/2 and α-estrogen receptor pathways. Additionally, GLP-1R activation enhances cardiac function post- myocardial infarction, even in obese models, supporting its cardioprotective and reparative potential [98].

## Therapeutic potential of GLP-1RAs in other rheumatologic and dermatologic disorders

The potential effects of GLP-1 RAs on PsD can be more clearly understood by examining how these drugs interact with the molecular and cytokine pathways common to PsD and other rheumatologic and dermatologic conditions, including rheumatoid arthritis (RA), osteoporosis (OP), osteoarthritis (OA), psoriasis (PsO), and hidradenitis suppurativa (HS) (Table 4).

**Table 4:** Shared molecular and cytokine pathways across psoriatic disease (PsD), rheumatoid arthritis (RA), osteoporosis (OP), osteoarthritis (OA), psoriasis (PsO) and hidradenitis suppurativa (HS)

Molecular/cytokine pathway	PsD	RA	OP	OA	PsO	HS	Function
NF-κB pathway	✓	✓	✓	✓	✓	✓	Master regulator of chronic inflammation
Th17 / Th1 pathway	✓	✓	✓	–	✓	✓	Adaptive immune pathways involved in autoimmunity and bone regulation
JAK/STAT pathway	✓	✓	–	✓	✓	✓	Key pathway downstream of cytokine receptors; synovial inflammation, keratinocyte activation, and immune dysregulation
PI3K/AKT pathway	✓	✓	✓	✓	✓	✓	Regulates cell survival, proliferation, inflammation, and bone metabolism
AMPK signaling	✓	✓	✓	✓	✓	✓	Metabolic regulator with anti-inflammatory and bone-protective roles
TNF	✓	✓	✓	✓	✓	✓	Key inflammatory cytokine; therapeutic target in PsO, PsD, RA, HS
IL-17	✓	✓	✓	–	✓	✓	Crucial in PsO and PsD; contributes to inflammation and bone loss
IL-23/IL-12	✓	✓	–	–	✓	✓	IL-23 drives Th17 axis; central in PsO and HS
IL-1β	✓	✓	✓	✓	✓	✓	Pro-inflammatory cytokine involved in joint and tissue damage
IL-6	✓	✓	✓	✓	✓	✓	Promotes osteoclastogenesis via RANKL; systemic inflammation
MMPs	✓	✓	✓	✓	✓	✓	Involved in ECM degradation and tissue remodeling
IL-36	✓	–	–	–	✓	✓	Highly expressed in PsO and HS
IFN-γ	✓	✓	✓	–	✓	✓	Modulates immune response and bone remodeling
IL-10 (anti-inflammatory)	↓	↓	↓	↓	↓	↓	Downregulated in inflammatory conditions

*AKT* known as the *PKB* Protein Kinase B; *AMPK* AMP-activated protein kinase; *IL* Interleukin; *IFN* Interferon; *JAK-STAT* Janus kinase-signal transducer and activator; *MMP* Matrix metalloproteinases; *NF-κB* Nuclear factor kappa B; *PI3K* Phosphatidylinositol 3-kinase; *TNF* Tumor necrosis factor

✓ Confirmed involvement; – Minor, unclear, or emerging involvement; ↓ Decreased expression

### Rheumatoid arthritis

Results from three experimental studies suggest that GLP-1RAs (lixisenatide, exenatide, and dulaglutide) show therapeutic potential for treating RA through similar mechanisms. The observed outcomes were particularly significant in fibroblast-like synoviocytes, key players in RA pathogenesis, with the following main effects: (1) inhibition of NF-κB nuclear translocation, leading to a decrease in the production of pro-inflammatory cytokines, including TNF, IL-6, IL-8, IL-1β, MCP-1, and High Mobility Group Box (HMGB)-1, all of which are involved in RA progression; (2) enhancement of mitochondrial function and attenuation of oxidative stress by reducing reactive oxygen species, thereby offering protection against the cellular damage characteristic of RA; (3) suppression of matrix metalloproteinases (MMPs), particularly MMP-3 and MMP-13, which are crucial mediators of cartilage degradation and joint inflammation [99–101]. In a prospective observational cohort study, Sullivan et al. evaluated liraglutide in 15 patients (11 with RA, 4 with PsA) with T2DM and active arthritis. Participants received liraglutide in addition to their ongoing immunosuppressive therapy. Over a 24-week period, responders exhibited significant improvements in disease activity score (DAS)28, swollen joint counts, body weight, and HbA1C, whereas non-responders showed no notable changes. Notably, weight reduction was significantly correlated with DAS28 improvement [102]. Gavazova et al. [103] conducted a prospective observational study evaluating 30 RA patients with obesity and T2DM treated with GLP-1 RAs for 6 months, without concomitant conventional or biologic disease-modifying anti-rheumatic drugs (DMARDs). The intervention was associated with reductions in inflammatory markers, clinical symptoms, and a 10% average weight reduction.

These preliminary findings suggest that GLP-1 RAs may have therapeutic potential in RA management improving disease activity, particularly in patients with metabolic comorbidities.

### Osteoporosis

The potential impact of GLP-1RAs on bone metabolism has been examined in three recent reviews [104–106]. GLP-1RAs beneficial effects on the bone are mediated by hormonal changes, such as increased calcitonin production [107], reduced sclerostin levels [108], and improved bone blood flow [105]. In preclinical models, these molecules improve bone mass [107], trabecular and cortical architecture [109–111], bone strength [112], and collagen content [111], but do not affect bone mineral density [113–115]. Additionally, GLP-1RAs can directly influence bone cells through GLP-1R expressed in osteoblasts, osteoclasts, and osteocytes [105].

GLP-1RAs, including liraglutide and exendin-4, activate several signaling pathways that regulate bone formation and metabolism. The GLP-1R/phosphatidylinositol 3-kinase (PI3K)/protein kinase B (AKT) pathway promotes osteoblast differentiation and reduces apoptosis [116, 117], while inhibitors like LY294002 partially suppress these effects, highlighting its role in bone health [118]. Additionally, GLP-1RAs activate the mitogen-activated protein kinase (MAPK) pathway (Erk1/2, JNK, p38), further supporting osteoblast differentiation and bone formation, with the inhibition of MAPK signaling blocking these protective effects [119]. The Wingless/Int-1 (Wnt)/β-catenin pathway is also activated by GLP-1RAs, enhancing osteogenic differentiation and inhibiting adipogenesis. Liraglutide increases cyclic adenosine monophosphate (cAMP) and β-catenin levels, boosting osteogenic activity [112]. Furthermore, the osteoprotegerin (OPG)/Receptor Activator of Nuclear Factor κB Ligand (RANKL) pathway is regulated by GLP-1RAs, increasing the OPG/RANKL ratio to inhibit osteoclast formation and promote bone formation by upregulating osteogenic genes like OC, COL1, Runx2, and ALP [120, 121].

A retrospective cohort study using real-world data from 1845 elderly patients with T2DM found that GLP-1 RAs use was associated with a significantly lower risk of developing OP, even after adjusting for multiple confounders including age, sex, BMI, smoking, and antihypertensive use. These findings suggest a potential bone-protective role of GLP-1 RAs in T2DM, warranting further investigation through RCTs [122].

### Osteoarthritis

GLP-1R signaling plays a key role in osteoarthritis (OA) by modulating inflammation, cartilage and bone metabolism, adipogenesis, and nociception. GLP-1RAs may influence OA through multiple pathways, not limited to weight reduction and improved glycemic control [123]. Emerging evidence suggests that GLP-1RAs may exert direct actions on joint homeostasis [91, 124–127]. They exhibit anti-inflammatory and chondroprotective effects by suppressing nuclear factor kappa B (NF-κB), protein kinase A (PKA)/cAMP Response Element-Binding protein (CREB), and MAPK pathways, reducing cytokine synthesis, oxidative stress, and endoplasmic reticulum stress. They also prevent cartilage degradation by inhibiting apoptosis and catabolic processes via PI3K/AKT and AMP-activated protein kinase (AMPK) signaling. In the bone, GLP-1 analogues enhance osteoblast activity and migration through Erk 1/2 MAPK, β-catenin, and Wnt/β-catenin pathways [128–136]. Additionally, they regulate adipocyte proliferation and differentiation and fatty acid degradation while exerting neuroprotective and analgesic effects by modulating cAMP, AMPK, and NF-κB pathways [129, 137, 138].

In vitro and animal models indicate that GLP-1RAs, such as liraglutide, mitigates cartilage degradation and downregulates inflammatory mediators like IL-6, prostaglandin (PG)E2, and nitric oxide [128]. Preclinical research indicates that intra-articular (IA) liraglutide attenuates synovial inflammation, decreases catabolic markers such as ADAMTS-5, and limits cartilage and bone damage across three experimental OA models, including MIA (mono-iodo-acetate), DMM (destabilization of the medial meniscus), and collagenase II-induced OA. Notably, the treatment preferentially preserved the inner tibial cartilage zone and was associated with a trend toward reduced osteophyte formation [139]. Complementing these observations, Meurot et al. compared IA liraglutide with dexamethasone or vehicle in the MIA model, revealing superior analgesic, anti-inflammatory, and structural outcomes with IA liraglutide. While dexamethasone only modestly reduced synovial inflammation, it failed to prevent cartilage deterioration, whereas liraglutide significantly enhanced pain thresholds, lowered synovial scores, and preserved cartilage integrity, with 60 µg doses notably reducing overall joint damage [140]. Together, these findings highlight liraglutide potential as a disease-modifying therapy that addresses both symptoms and structural joint preservation in OA.

Despite promising preclinical results, T2DM clinical trials have shown variable outcomes [141]. In particular, a randomized controlled trial investigated liraglutide in people with overweight/obesity and knee OA; after initial weight loss through diet, participants received liraglutide or placebo for one year. Liraglutide led to modest weight loss and improved physical function but did not significantly reduce knee pain or increase daily physical activity. The findings suggest its pain-relief benefits may depend on the extent of weight loss achieved [142, 143]. A prospective observational study by Zhu et al. investigates the potential role of GLP-1RAs as DMARDs in 1807 patients with knee OA and T2DM. GLP-1RA users showed greater weight loss, lower knee surgery incidence, reduced pain, slower cartilage loss compared to the control group. Symptom-relieving medication also decreased post-treatment. The findings suggest that GLP-1RAs may have disease-modifying effects in knee OA, primarily mediated by weight reduction [145]. In a 68-week randomized, placebo-controlled trial conducted by Bliddal et al. involving 407 individuals with obesity and knee OA, treatment with semaglutide resulted in significantly greater reductions in body weight (− 13.7% *vs*. − 3.2%) and knee pain (− 41.7 *vs*. − 27.5 points on the WOMAC scale) compared to placebo. Notable improvements in physical function, as assessed by the SF-36 score (+ 12.0 *vs*. + 6.5), were also reported [146].

The efficacy of other GLP-1RAs, including semaglutide and dual GLP-1/GIP agonist tirzepatide, in OA treatment is still under investigation. Given their dual benefits in obesity and inflammation regulation, GLP-1RAs medications hold potential as disease-modifying therapies for OA. However, further research is needed to confirm their effectiveness and long-term impact [144].

### Psoriasis

As previously discussed, chronic low-grade inflammation is a common pathological feature of both obesity and PsO, contributing to disease burden. Additionally, weight loss is linked to decreased PsO severity and enhanced quality of life [147]. In a RCT by Lin et al. 12 patients with psoriasis and T2DM underwent liraglutide treatment. Compared to the control group, they exhibited greater improvement in Psoriasis Area Severity Index (PASI) and Dermatology Life Quality Index (DLQI). Histopathological evaluation demonstrated a reduced expression of inflammatory cytokines, such as IL-17, IL-23, and TNF, in the psoriatic skin of patients receiving liraglutide, suggesting a potential link to skin lesion improvement [148]. In a prospective cohort study by Nicolau et al. [149], liraglutide was shown to be a safe and effective treatment for both weight reduction and psoriatic lesion improvement in a cohort 20 patients with PsO and obesity. Psoriatic arthritis was present in 30% of patients but no evaluation of articular disease activity was made. Significant decreases were observed in BMI, C-reactive protein, homocysteine, ferritin, and plasma cortisol levels, alongside significant improvements in PASI and DLQI. Notably, weight loss did not correlate with inflammatory parameters or PASI, suggesting that the improvement in skin lesions occurred independently of weight reduction. The interaction between GLP-1RAs and the immune system in inflammatory conditions was investigated by Hogan et al. in 2 patients with PsO and T2DM. After 6 weeks of treatment, a reduction in PASI score was observed, along with an increase in circulating invariant Natural Killer (iNK) T cells and a decrease in their number within the psoriatic plaque. Additionally, GLP-1RAs induced a dose-dependent inhibition of iNK T cell cytokine secretion, without affecting cytolytic degranulation in vitro [150]. In a prospective cohort study, liraglutide was found to improve PASI and DLQI in patients with both PsO and T2DM. This GLP-1 analogue also exhibited immunomodulatory properties, enhancing the number of circulating iNKT cells and reducing the proportion of TNF-producing monocytes [151]. Comparable immunological outcomes were observed in a prospective case series study involving 7 patients with PsO and T2DM treated with exenatide or liraglutide. Results demonstrated improved PASI scores, alongside a reduction in dermal γδ T-cell numbers and IL-17 expression, as well as a decrease in epidermal thickness [152]. In a case report, semaglutide was proved highly effective against severe psoriasis in a T2DM patient showing improvement in PASI, DLQI as well as in HbA1c and BMI [153]. A 12-week RCT in 31 obese patients with T2DM and PsO assessed the impact of semaglutide compared to metformin on PsO activity. Patients receiving semaglutide showed significant improvements in PASI and DLQI, along with reductions in serum and LDL cholesterol levels, compared with controls. Although a decrease in inflammatory parameters was observed in both groups, statistical significance was achieved only for CRP and IL-6 in the semaglutide arm. During the study period, three participants discontinued treatment: two due to gastrointestinal adverse effects (nausea and vomiting) and one due to PsO exacerbation [154].

In contrast, study on liraglutide show variable outcomes. In a randomized placebo-controlled trial on 20 obese, glucose-tolerant patients with plaque PsO no significant change in PASI, DLQI and high sensitive CRP were observed after 8 weeks treatment with liraglutide compared to placebo group [155]. These findings conflict with the results of a small prospective open-label cohort of patients with PsO and T2DM, where 10 weeks of liraglutide treatment resulted in a significant reduction in median PASI and median DLQI. Additionally, circulating iNKT cells increased from 0.13% to 0.40% of T lymphocytes [151]. These results are in line with a prospective cohort study in seven patients with psoriasis and T2DM, in which 12 weeks of liraglutide treatment significantly decreased PASI and DLQI, improved glycemic control, reduced BMI and waist circumference, and produced histological skin improvements, including reduced epidermal thickness [156].

### Hidradenitis suppurativa

Hidradenitis suppurativa (HS) is a chronic inflammatory skin disorder characterized by follicular hyperkeratosis and epithelial hyperplasia, leading to follicular obstruction and immune activation. This process is mediated by pro-inflammatory cytokines like IL-1β and TNF, and Th1 and Th17 mediators including IFNγ and IL-17 [157]. This condition is closely associated with metabolic abnormalities, including obesity, diabetes, and dyslipidemia [158]. Obesity-related inflammatory imbalance elicits follicular occlusion and HS progression through multiple molecular mechanisms [147]. GLP1-RAs demonstrate significant potential as an adjunct therapy for HS, addressing both the metabolic and inflammatory aspects of the condition [158]. Nicolau et al. investigated the impact of liraglutide 3 mg in 14 obese patients with HS, finding significant reductions in BMI, waist circumference, CRP, homocysteine, and plasma cortisol after 3 months treatment. Additionally, there was an improvement in lesion severity, as assessed by the Hurley Staging System and DLQI [159]. In a retrospective study, Lyons et al., evaluated the effects of semaglutide in 30 obese patients with HS, reporting reductions in weight, BMI, and metabolic markers such as HbA1c, along with improvements in DLQI and a decreased frequency of flares [160].

## Conclusion

The interrelationship between PsD and its metabolic comorbidities – namely obesity, T2DM, and CV disease – is increasingly recognized as a critical determinant of patient outcomes requiring multidisciplinary management. In this context, GLP-1RAs emerge as a promising class of drugs that could address both metabolic and inflammatory dimensions of PsD in adjunct to the necessary lifestyle interventions. Their established benefits in weight reduction, glycemic control, and cardiovascular protection make them particularly suitable for this high-risk patient population. Additionally, their potential anti-inflammatory properties mediated by inhibition of key signaling pathways, could align well with the immunopathogenesis of PsD, as suggested by preclinical and clinical evidence supporting their broader immunomodulatory across a spectrum of rheumatological and dermatological conditions.

It has to be underlined that, while current evidence of GLP-1RAs efficacy on PsD-related comorbidities relies on solid systematic reviews, RCTs, and large cohort studies, their potential role in rheumatologic disorders, particularly in PsD, remains largely unexplored. As summarized in Table S1, the level of evidence is still low, derived mainly from in vitro studies, animal models, and small patient cohorts, with only limited data from RCT available. Similarly, the specific safety profile of GLP1-RAs in PsD is yet to be established, since current evidence is confined to small cohorts of patients with PsO undergoing liraglutide. In these studies, no major adverse events have been reported; however, gastrointestinal adverse events such as nausea, loss of appetite and constipation were observed [155, 156]. In addition, the heterogeneity in study designs, patient populations, condition, and outcome measures from the studies herein analyzed further complicates direct comparisons and limits the possibility of drawing solid conclusions.

Recent RCTs are beginning to explore the dual impact of metabolic and inflammatory modulation in PsD, although no results have yet been published, representing important future perspectives in this field. In PsA, the phase 3b TOGETHER-PsA trial (NCT06588296) assesses the combined use of ixekizumab and tirzepatide in patients with active disease and concomitant overweight or obesity, while the phase 4 TOGETHER AMPLIFY-PsA study (NCT06864026) examines the effectiveness of adding tirzepatide to ixekizumab in clinical practice over 12 months. In parallel, a phase 4 RCT (NCT07111494) will compare GLP-1RAs with nutritional counseling in PsA patients with obesity and T2DM, incorporating both clinical and patient-reported outcomes. In PsO, ongoing studies include phase 3 and 4 trials of tirzepatide combined with ixekizumab (NCT06588283; NCT06857942), as well as investigations of semaglutide (NCT06475586), liraglutide, and exenatide (NCT01687582) in patients with T2DM. Collectively, these investigations aim to clarify whether GLP-1RAs may exert immunometabolic effects relevant to PsD pathogenesis and treatment.

In conclusion, GLP-1RAs represent a novel, multifaceted approach to PsD management. While further RCTs are essential to confirm their efficacy and safety in PsD-specific contexts, preliminary evidence underscores their promise in reshaping the treatment landscape by simultaneously targeting systemic inflammation and metabolic dysregulation.

## Supplementary Information

Below is the link to the electronic supplementary material.

Supplementary file1 (DOCX 69 kb)

## Data Availability

No datasets were generated or analysed during the current study.
