# Supplementary material for: The potential role of GLP-1 receptor agonists in the management of psoriatic disease: a scoping review
Source: Inflamm Res. 2025 Nov 21;74(1):167. doi: 10.1007/s00011-025-02140-2 (PMC12634801; doi:10.1007/s00011-025-02140-2)
Supplement: Supplementary file 1 — Supplementary file1 (DOCX 69 kb) [file 11_2025_2140_MOESM1_ESM.docx]

**Table S1.** Levels of evidence supporting GLP-1RA use in rheumatologic conditions.

| Condition | Authors | Type of evidence | Level of evidence |
| --- | --- | --- | --- |
| Rheumatoid arthritis | *Sullivan et al.[104]* | Prospective observational study | 2b |
|  | *Gavazova et al.[105]* | Prospective observational study | 2b |
|  | *Tao et al.[101]* | In vitro study | 5 |
|  | *Zheng et al.[102]* | In vitro study | 5 |
|  | *Du et al. [103]* | In vitro study | 5 |
| Osteoporosis | *Bunck et al. [115]* | Prospective, randomized, controlled clinical trial | 2b |
|  | *Li et al. [116]* | Randomized, parallel-group clinical trial | 2b |
|  | *Gilbert et al. [117]* | Randomized, double-blind, controlled clinical trial, fase III | 2b |
|  | *Chen et al.[124]* | Retrospective observational cohort study | 4 |
|  | *Yamada et al.[109]* | Animal research | 5 |
|  | *Kim J et al. [110]* | Animal research | 5 |
|  | *Nuche-Berenguer et al.[111]* | Animal research | 5 |
|  | *Sun et al. [112]* | Animal research | 5 |
|  | *Mansur et al. [113]* | Animal research | 5 |
|  | *Meng et al. [114]* | Animal research | 5 |
|  | *Xie et al.[121]* | Animal research | 5 |
|  | *Yang et al.* | Animal research | 5 |
|  | *Wu et al.* | Animal research | 5 |
|  | *Xie et al.[118]* | Animal research | 5 |
|  | *Nuche-berenguer et al. [122]* | Animal research | 5 |
|  | *Ma et al. [123]* | Animal research | 5 |
|  | *Mabilleau et al. [107]* | Non-systematic review of results | 5 |
|  | *Xie et al.[108]* | Non-systematic review of results | 5 |
| Osteoarthritis | *Ryan et al. [125]* | Randomized, placebo-controlled, multicenter clinical trial | 2b |
|  | *Aronne et al. [93]* | Randomized, placebo-controlled, multicenter clinical trial | 2b |
|  | *Gudbergsen et al. [145]* | Prospective, randomized, placebo-controlled clinical trial | 2b |
|  | *Bartholdy et al.[146]* | Secondary analysis of a randomized, placebo-controlled clinical trial | 2b |
|  | *Bliddal et al. [149]* | Prospective, double-blind, randomized, placebo-controlled clinical trial | 2b |
|  | *Zhu et al. [148]* | Prospective observational cohort study | 3b |
|  | *Ryan et al. [125]* | Non-systematic review of results | 5 |
|  | *Chowdhury et al. [126]* | Non-systematic review of results | 5 |
|  | *Jensen et al. [85]* | Non-systematic review of results | 5 |
|  | *Jensterle et al. [129]* | Non-systematic review of results | 5 |
|  | *Que et al. [130]* | Animal research | 5 |
|  | *Berenbaum et al. [142]* | Animal research | 5 |
|  | *Chen et al.[132]* | Animal research | 5 |
|  | *Mei et al. [133]* | In vitro study | 5 |
|  | *Shiraishi et al. [134]* | In vitro study | 5 |
|  | *Wan et al. [135]* | Animal research | 5 |
|  | *Meurot et al. [136]* | Non-systematic review of results | 5 |
|  | *Li et al. [137]* | In vitro study | 5 |
|  | *Tong et al. [138]* | In vitro study | 5 |
|  | *Gong et al. [139]* | Animal research | 5 |
|  | *Que et al. [130]* | Animal research | 5 |
|  | *Meurot et al. [143]* | Animal research | 5 |
|  | *Hogan et al. [144]* | In vitro study | 5 |
|  | *Jamal et al. [147]* | Non-systematic review of results | 5 |
| Psoriasis | *Lin et al. [151]* | Prospective, randomized, controlled clinical trial | 2b |
|  | *Petković-Dabić et al. [157]* | Prospective, randomized, controlled clinical trial | 2b |
|  | *Faurschou et al. [158]* | Prospective, randomized, placebo-controlled clinical trial | 2b |
|  | *Nicolau et al. [152]* | Open-label trial | 4 |
|  | *Hogan et al.[153]* | Case-series | 4 |
|  | *Buysschaert et al. [155]* | Case-series | 4 |
|  | *Costanzo et al. [156]* | Case-report | 5 |
|  | *Vilarrasa et al.* | Non-systematic review of results | 5 |
|  | *Ahern et al. [154]* | Prospective, single-arm, interventional study | 5 |
|  | *Xu et al. [159]* | Prospective, single-arm, interventional study | 5 |
| Hidradenitis suppurativa | *Krajewski et al. [161]* | Systematic review of (homogeneous) cohort studies | 2a |
|  | *Nicolau et al. [162]* | Studio prospettico, single-arm, interventional study | 4 |
|  | *Lyons et al. [163]* | Retrospective observational study | 4 |
